# Longitudinal Comparison of Enzyme- and Laser-Treated Intervertebral Disc by MRI, X-Ray, and Histological Analyses Reveals Discrepancies in the Progression of Disc Degeneration: A Rabbit Study

**DOI:** 10.1155/2016/5498271

**Published:** 2016-05-10

**Authors:** Marion Fusellier, Pauline Colombier, Julie Lesoeur, Samy Youl, Stéphane Madec, Olivier Gauthier, Olivier Hamel, Jérôme Guicheux, Johann Clouet

**Affiliations:** ^1^Institut National de la Santé et de la Recherche Médicale (INSERM) UMRS 791, Laboratoire d'Ingénierie Ostéo-Articulaire et Dentaire (LIOAD), Group STEP “Skeletal Tissue Engineering and Physiopathology”, School of Dental Surgery, 44042 Nantes, France; ^2^Department of Diagnostic Imaging, CRIP, National Veterinary School (ONIRIS), 44307 Nantes, France; ^3^Odontology Faculty, University of Nantes, 44093 Nantes, France; ^4^Department of Experimental Surgery, CRIP, National Veterinary School (ONIRIS), 44307 Nantes, France; ^5^Medicine Faculty, University of Nantes, 44093 Nantes, France; ^6^PHU4 OTONN, University Hospital of Nantes, 44093 Nantes, France; ^7^Department of Pharmacy, University Hospital of Nantes, 44093 Nantes, France; ^8^Pharmacy Faculty, University of Nantes, 44093 Nantes, France

## Abstract

Regenerative medicine is considered an attractive prospect for the treatment of intervertebral disc (IVD) degeneration. To assess the efficacy of the regenerative approach, animal models of IVD degeneration are needed. Among these animal models, chemonucleolysis based on the enzymatic degradation of the Nucleus Pulposus (NP) is often used, but this technique remains far from the natural physiopathological process of IVD degeneration. Recently, we developed an innovative animal model of IVD degeneration based on the use of a laser beam. In the present study, this laser model was compared with the chemonucleolysis model in a longitudinal study in rabbits. The effects of the treatments were studied by MRI (T2-weighted signal intensity (T2wsi)), radiography (IVD height index), and histology (NP area and Boos' scoring). The results showed that both treatments induced a degeneration of the IVD with a decrease in IVD height and T2wsi as well as NP area and an increase in Boos' scoring. The enzyme treatment leads to a rapid and acute process of IVD degeneration. Conversely, laser radiation induced more progressive and less pronounced degeneration. It can be concluded that laser treatment provides an instrumental in vivo model of slowly evolving IVD degenerative disease that can be of preclinical relevance for assessing new prophylactic biological treatments of disc degeneration.

## 1. Introduction

Low back pain (LBP) is an extremely frequent symptom that affects up to 20% of individuals aged from 30 to 64 years [1], and 70–85% of all people suffer from back pain at some time in life [2]. It is therefore becoming a major public health concern (approximately 650 million individuals are affected in the world) with an increasing socioeconomic cost in aging populations. In 40% of patients, this low back pain is thought to stem from intervertebral disc degenerative disease (DDD), also called discogenic LBP [3]. DDD is primarily managed by pharmacological treatments and, if unsuccessful, by surgical procedures (spine fusion or disc prosthesis) whose goal is to relieve pain. Nevertheless, these surgical treatments are particularly invasive and have limitations, despite significant improvements, notably the development of new minimally invasive routes. Given the limitations of these therapies, regenerative medicine has recently been increasingly considered as an attractive prospect for the treatment of DDD [4].

Spontaneous degeneration of the intervertebral disc (IVD) involves mechanical processes and aging that lead to the degradation of the extracellular matrix of the Nucleus Pulposus (NP). This deterioration in turn induces dehydration of the NP that ultimately decreases IVD resistance to mechanical constraints. IVD degeneration is classically investigated, in vivo, using magnetic resonance imaging (MRI) and radiography. A decrease in the T2-weighted signal intensity (T2wsi) and a later decrease in the IVD height index are observed on MRI and X-rays, respectively. At the same time, histological analyses of the degenerated IVD usually show a cell density decrease, the loss of a clearly delimited boundary between NP and annulus fibrosus (AF), and an associated increase in the histological Boos' scoring. This Boos' scoring closely reflects the histological state of the degenerated IVD on the basis of criteria including cell density, granular changes, tear and cleft formation, and mucus degeneration.

To demonstrate the relevance of innovative regenerative medicine approaches, and before transposing the approach to humans, animal models of DDD that closely mimic the slowly evolving IVD degeneration observed in humans are required. In this context, a relevant animal model should include the following features: (1) an animal tall enough to allow introduction of therapeutic agents directly into the disc; (2) an animal that is easy to obtain and inexpensive; (3) DDD developed in a progressive time-dependent manner; and (4) DDD histologically close to spontaneous degeneration. Many models have been developed so far, but none gather the required features. Among animal models described in recent years [5, 6], two subgroups can be defined: models with spontaneous and experimentally induced disease.

The spontaneous disease models are probably among the most relevant models since they are expected to closely mimic the physiopathological process of DDD. Unfortunately, they often take too long to evolve to make them appropriate models for experimental studies.

Numerous experimentally induced DDD animal models have been used in preclinical studies and are commonly categorized in three groups: mechanical, physical, and chemical models. Among the mechanical models, mechanical overload has been induced by multiple procedures [7–14]. Most of the time, while animals used (rabbits or dogs) are large enough to consider regenerative therapies via percutaneous approaches, these models remain time-consuming, painful, ethically debatable, and expensive, particularly the canine models.

Physical models usually combine various methods of inducing disc lesions including AF disruption [15] and NP aspiration [16]. The scalpel-induced disruption of AF presents the disadvantage of being unreliable because it remains difficult to remove the same amount of AF material. NP aspiration consists of needle-assisted removal of the NP content. Although it can be used for biomechanical studies, the aspiration process is far from reflecting the progressivity and complexity of IVD degeneration.

The other commonly used animal model is chemonucleolysis [17–24], based on the intra-NP injections of proteolytic enzymes (hyaluronidase, papain, or chondroitinase ABC). These enzymes induce a chemical degradation of the NP extracellular matrix, particularly proteoglycan and collagens, which are essential for the biomechanical properties of the IVD [17]. The major limitation of chemonucleolysis is that it induces an acute degenerative process, despite the fact that it can be tuned by varying the concentration of enzyme injected into the disc. In addition, deleterious effects caused by the persistence of enzyme and the presence of metabolites could be considered as a limitation to investigate regenerative medicine approaches in these models.

In this context and considering the numerous limitations of currently available animal models, an innovative approach has been proposed using a laser radiation treatment [25]. Our preliminary data [25] demonstrated that laser treatment induced degenerative changes in rabbit IVD, making this model a promising tool to help validate cell-based strategies for the prevention and treatment of early stages of IVD degeneration. However, to further assess the relevance of laser radiation as a model of DDD, we sought to compare this model with the chemonucleolysis model, notably to evaluate the ability of laser to induce slowly evolving DDD.

In the present study, the longitudinal effects of laser and enzyme treatments were assessed for a 3-month period in 1-year-old rabbits. To evaluate the ability of laser to induce progressive DDD, degenerative changes of the IVD were followed using MRI (T2-weighted signal intensity), X-rays (IVD height), and histological Boos' scoring.

## 2. Materials and Methods

### 2.1. Ethical Aspects and Animals

All animal handling and surgical procedures were conducted according to European Community guidelines for the care and use of laboratory animals (DE 86/609/CEE). The Pays de la Loire ethics committee approved the animal study protocol (agreement CEEA.2012.16 and CEEA.2012.181).

Thirteen healthy female New Zealand white rabbits (age, 1 year; weight, 2.5–3.5 kg; Grimaud Frères, Roussay, France) were used for the study. Three of the 13 rabbits were used as controls and no surgical treatment was performed on them.

### 2.2. Surgical Procedure

A preoperative MRI was performed to eliminate spontaneous degenerated IVDs from the study. IVD lesions were induced under general anesthesia and fluoroscopic guidance following two methods: enzyme and laser treatment. The animals were tranquilized with an intramuscular injection of ketamine (15–20 mg/kg) and xylazine (2 mg/kg) and then put under gaseous anesthesia (NO (0.5 L/min)/O_2_ (0.5 L/min)/isoflurane (2%)). A longitudinal mid-line skin incision was made from the xiphoid to the pelvic rim. The anterior side of the vertebral column from L1 to L6 was exposed via the transperitoneal approach.

Disc levels were identified under fluoroscopic guidance. Four IVDs (L2-L3, L3-L4, L4-L5, and L5-6) were used for the experiment, and the lesions were randomized according to the protocol summarized in Table 1.

The enzyme treatment consists in injecting 20 *μ*L of hyaluronidase (50 *μ*g/*μ*L in PBS 1x; 37.5–150 IU/mL; ref H4272; Sigma-Aldrich, St. Louis, MO, USA), using 25 Ga needle, at the lumbar IVD according to the modalities previously described [17].

Laser treatment was performed using a KaVo GENTLEray 980® diode laser as previously described [25]. Briefly, after AF puncture with a 25-gauge needle to allow the insertion of the 300 *μ*m laser fiber, the end of the fiber was guided into the NP under fluoroscopic guidance. The parameters used were pulse sequence mode with 1.6 W pulse output, pulse length of 200 ms, pulse spacing of 200 ms, and duration of 40 s. These parameters were determined by preliminary studies [25].

The abdominal white line and skin were closed in layers with resorbable sutures.

### 2.3. MRI and X-Ray Scanning Procedure

For imaging procedures, the rabbits were tranquilized with an intramuscular injection of dexmedetomidine (100 *μ*g/kg) and ketamine (15–20 mg/kg). MRI and X-rays were always performed on each rabbit at day 0 to exclude spontaneously degenerated IVDs from a further surgical procedure and then at days 7, 30, 60, and 90.

An MRI of the entire lumbar spine was performed on a 1.0 T MR scanner (Magnetom Harmony, Siemens Medical Solutions, Erlangen, Germany) using a standard body coil to obtain T2-weighted images (TR, 5000 ms; TE, 111 ms) with the following parameters: matrix, 400 × 200; field of view, 200 × 100 mm; and slice thickness, 3 mm with no interslice gap.

Plain radiographs of the spines were taken using a radiograph machine (Convix 80 generator and Universix 120 table) from Picker International (Uniontown, OH, USA). Coronal and sagittal plain radiographs of the spines were taken with a collimator-to-film distance of 100 cm, exposure of 100 mAs, and penetration power of 48 kVp.

### 2.4. Image Analysis

The image data were analyzed with Osirix software 3.9 (Osirix Foundation, Geneva, Switzerland).

T2-weighted signal intensity (T2wsi) was determined on MRI images on a midsagittal slice. T2wsi is measured by the ratio of NP mean weighted signal intensity divided by the spinal cord signal measured at each time point.

The index of IVD height was measured on X-rays. This index represents the mean of the ventral and the dorsal height of the disc divided by mean of the ventral and dorsal length of the adjacent vertebra. The results were expressed as relative IVD height decrease.

### 2.5. Histological Analysis

At days 0, 7, and 90, three rabbits were sacrificed by intramuscular injection of ketamine (15–20 mg/kg) followed by an intravenous injection of sodium pentobarbital (1.2 g/kg). For ethical reasons, we chose to limit the number of animals sacrificed and no sacrifice was performed at days 30 and 60. The lumbar IVDs were then collected from four consecutive levels (L2-L3 to L5-L6). IVDs were dissected and fixed in 10% neutral buffered formalin for 1 week and decalcified for 24 h in Decalcifier II® (Surgipath, Richmond, IL, USA). After dehydration and incubation with Histosol® (Shandom, Brussels, Belgium), specimens were embedded in paraffin and sectioned into 3 *μ*m slices. For histological analysis, 3 *μ*m thick paraffin sections were deparaffinized using toluene, rehydrated through a graded series of ethanol, and rinsed in distilled water. Sections were stained with hematoxylin phloxine safran (HPS) and 0.1% Alcian blue-PAS (Sigma-Aldrich, St. Louis, MO, USA) as previously described [26].

For each IVD, using HPS stainings, NP area measurements were taken using NDP view2 software (Hamamatsu Photonics) and expressed in square millimeters.

Histological sections were also analyzed using a modified Boos' scoring [27] specifically designed to characterize the histomorphology of the IVD and to evaluate the degenerative changes in the extracellular matrix of the NP. Briefly, this Boos' scoring was based on the analysis of four criteria: decrease in cell density, granular changes, tear and cleft formation, and mucus degeneration. Some parameters were ranked from 0 to 4 (granular and mucus degeneration) and others were ranked from 0 to 5 (decrease in cell density, and tear and cleft formation) depending on the intensity of the parameters tested (0, lowest; 4 or 5, highest). Three independent investigators who had expertise in reading histological slides performed a blind evaluation of histological samples.

### 2.6. Statistical Analysis

Statistical analysis was performed with R software. IVD height, T2wsi, Boos' scoring, and NP area were assessed for statistically significant differences using the Tukey test after an analysis of variance test. The significance level was set at 0.05.

## 3. Results

### 3.1. MRI T2-Weighted Signal Intensity Changes

**Figure 1:**
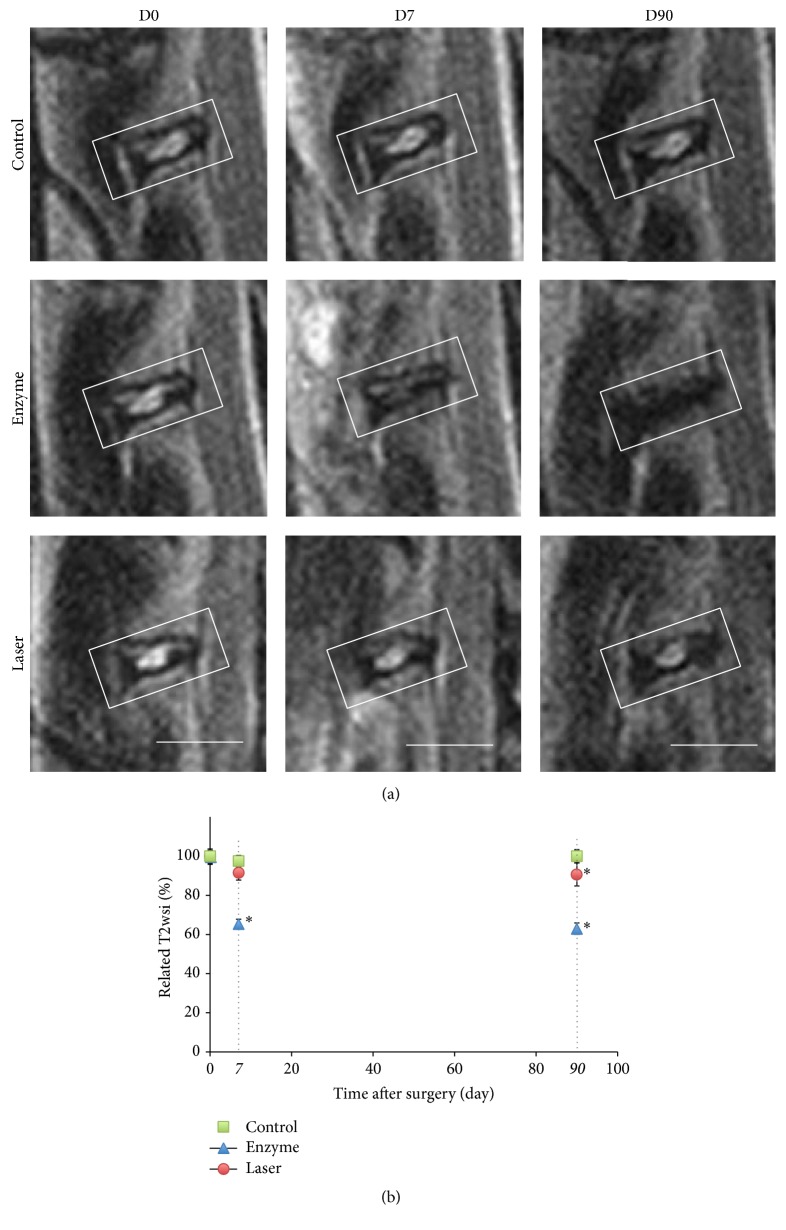
MRI images (T2-weighted midsagittal) and analysis of rabbit lumbar spines. Intervertebral discs of 1-year-old rabbits were treated according to either the enzyme technique (enzyme) or the laser procedure (laser) as described in Materials and Methods. Untreated IVDs were used as an internal control. (a) After the indicated times, a magnetic resonance imaging (MRI) scan of the spine was performed. Representative MRI scans are shown. Bar: 1 cm. (b) T2-weighted signal intensity was measured after the indicated times as described in Materials and Methods. ^*∗*^ *P* < 0.05 as compared with day 0. Values are expressed as mean ± SEM.

To analyze the degenerative changes of rabbit intervertebral discs, we first measured the T2wsi changes using MRI. For the enzyme-treated discs, the T2wsi image showed an intense and significant decrease from day 7 (35%  ± 2.35%) compared to control (2.5%  ± 3%) and the laser group (8.5%  ± 3.8%). This decrease was more progressive and delayed in the laser group in which it became significant only at day 90 compared with the control group (Figure 1).

### 3.2. IVD Height Changes

**Figure 2:**
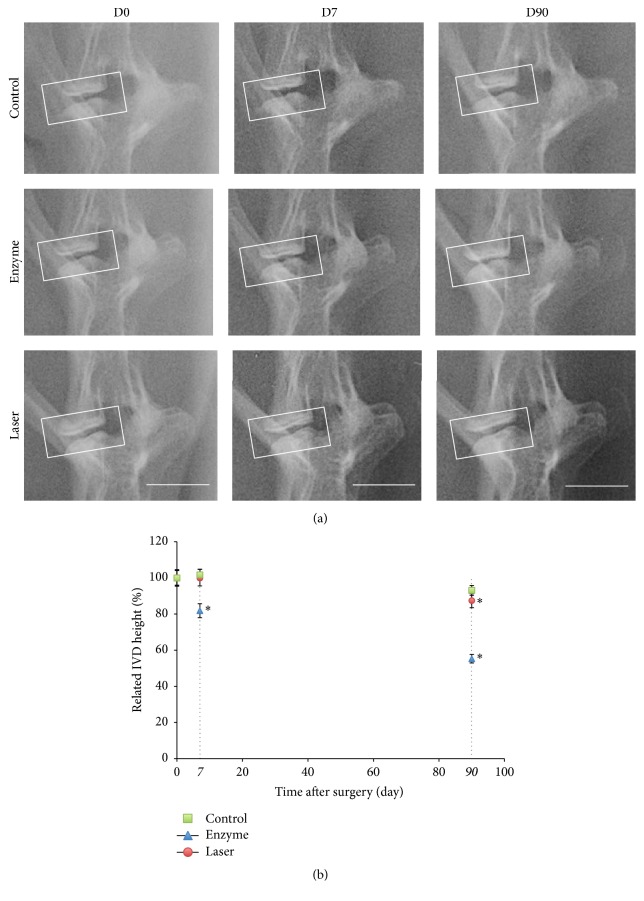
X-ray images and analysis of rabbit lumbar spines. Intervertebral discs of 1-year-old rabbits were treated according to either the enzyme technique (enzyme) or the laser procedure (laser) as described in Materials and Methods. Untreated IVDs (control) were used as an internal control. (a) After the indicated times, X-ray images of spines were taken. Representative X-ray images are shown. Bar: 1 cm. (b) Decrease in IVD height (%) was measured after the indicated times as described in Materials and Methods. ^*∗*^ *P* < 0.05 as compared with day 0. Values are expressed as percentage of decrease ± SEM.

To further analyze the IVD degenerative process, we then measured the changes in disc height using X-rays. The changes observed confirm the modification observed on MRI. Indeed, for both treatments, significant and gradual IVD narrowing as compared with untreated discs was observed (Figure 2). Ninety days after surgery, 45% and 13% (±SEM) decrease in IVD height were observed in enzyme- and laser-treated discs, respectively (Figure 2). A time-course analysis also revealed that the IVD height decrease occurred more quickly with the hyaluronidase treatment compared to with the laser treatment. Indeed, the early decrease in the IVD height was significantly different between controls and enzyme-treated discs as early as day 7, while in laser-treated discs, a significant decrease in IVD height was not observed before day 90.

### 3.3. Histological Analysis and Boos' Scoring

**Figure 3:**
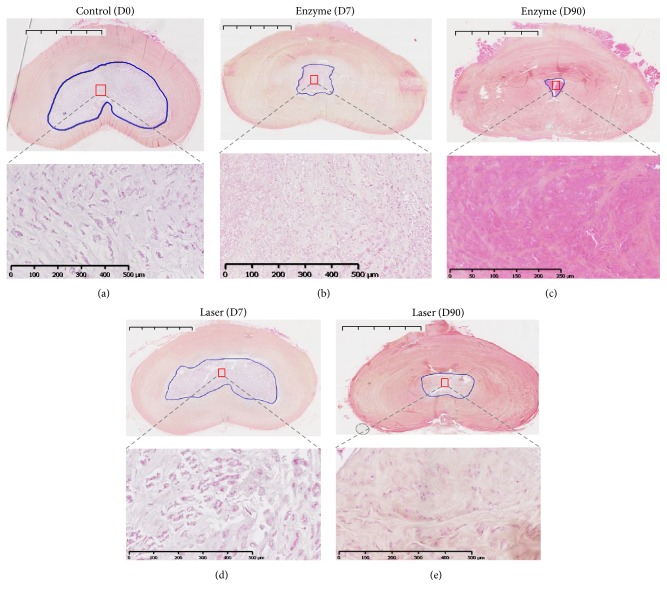
Histological analysis of rabbit intervertebral discs (IVDs) using hematoxylin phloxine safran (HPS) staining. IVDs of 1-year-old rabbits were treated according to either the enzyme technique (enzyme) or the laser procedure (laser) as described in Materials and Methods. IVDs were processed for histological analysis at days 0 (untreated IVD from two rabbits as internal control) and 7 and 90 days after treatment as described in Materials and Methods. Representative hematoxylin phloxine safran is shown at days 0, 7, and 90. For clarity reasons and regarding similarities in follow-up, only the control at day 0 is illustrated. Bar: 5 mm for macroscopic section image and 500 *μ*m for magnification image.

**Figure 4:**
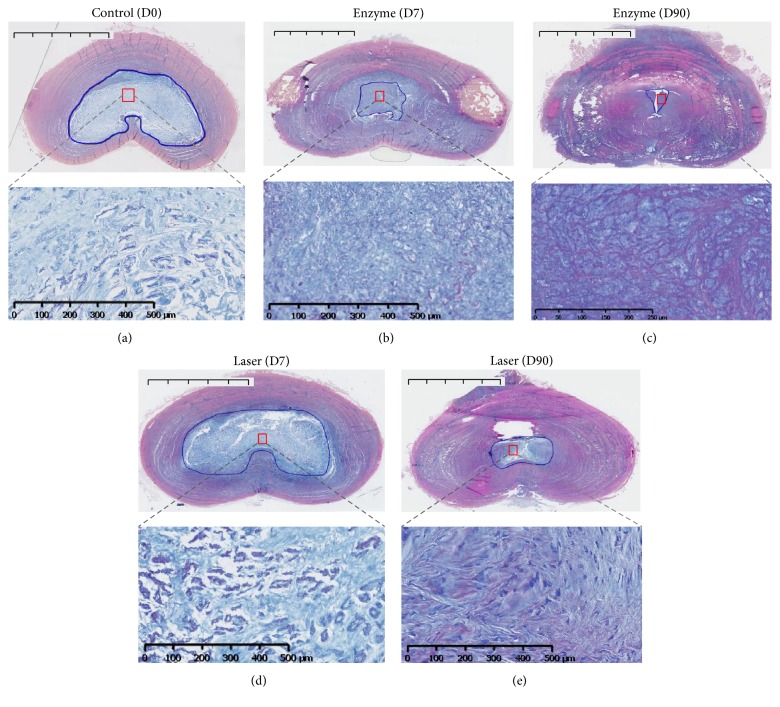
Histological analysis of rabbit intervertebral discs (IVDs) by Alcian blue/PAS staining (BA-PAS). IVDs of 1-year-old rabbits were treated according to either the enzyme technique (enzyme) or the laser procedure (laser) as described in Materials and Methods. IVDs were processed for histological analysis at days 0 (untreated IVD from two rabbits as internal control) and 7 and 90 days after treatment as described in Materials and Methods. Representative Alcian blue/PAS staining is shown at days 0, 7, and 90. For clarity reasons and regarding similarities in follow-up, only the control at day 0 is illustrated. Bar: 5 mm for macroscopic section image and 500 *μ*m for magnification image.

**Figure 5:**
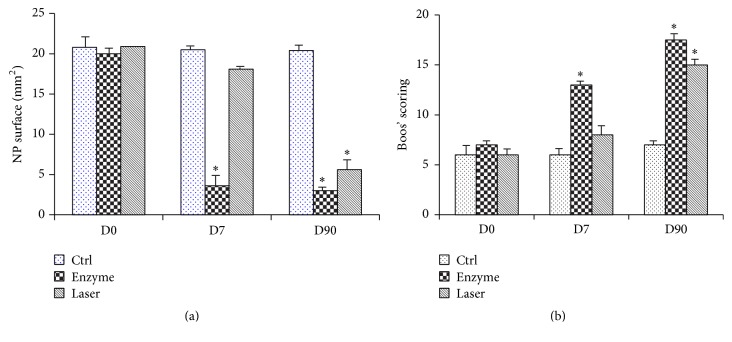
Evaluation of NP area and Boos' scoring for rabbit intervertebral discs (IVDs). IVDs of 1-year-old rabbits were treated according to either the enzyme technique (enzyme) or the laser procedure (laser) as described in Materials and Methods. Untreated IVDs from two rabbits were used as an internal control. NP area (a) and Boos' scoring (b) were determined for the different conditions at 0, 7, and 90 days as described in Materials and Methods. ^*∗*^ *P* < 0.05 compared to control. Values are expressed as mean ± SEM.

To further address whether the experimentally induced degeneration observed on MRI and radiological changes were correlated with tissue changes, we then performed histological analyses on histological stainings (HPS and BA-PAS) (Figures 3 and 4). To quantitatively assess tissue changes, the NP surface was measured and assessed with Boos' scoring (Figure 5).

Regarding HPS stainings, compared to the control condition at day 0 (Figure 3(a)), the injured IVD analysis after enzyme treatment showed a decrease in cell density at days 7 and 90 (Figures 3(b) and 3(c)). Interestingly, this decrease occurred early in the enzyme-treated group beginning at day 7 (Figure 3(b)), in contrast to the laser group in which the cell density gradually decreased from day 0 to day 90 (Figures 3(d) and 3(e)). Regarding BA-PAS staining, the extracellular matrix of the NP in the control condition demonstrated no notable changes (Figure 4(a)). Conversely, an alteration of the extracellular matrix was observed in the enzyme (Figures 4(b) and 4(c)) and laser (Figures 4(d) and 4(e)) groups characterized by mucoid and granular changes that reflect extracellular matrix degeneration. Compared with changes observed in the enzyme condition (Figures 4(b) and 4(c)), the mucoid and granular changes in the laser condition (Figures 4(d) and 4(e)) were less pronounced. Moreover, the mucoid and granular changes appeared as early as day 7 in the enzyme condition (Figure 4(b)).

The degenerative changes of the NP induced by enzyme and laser treatments were finally quantitatively assessed by measuring the NP surface from the HPS section (Figure 5(a)). Compared to controls, the enzyme treatment induced a drastic decrease in the NP area as early as day 7, compared with controls (mean, 3.6 versus 20.5) but also compared with laser treatment (mean, 3.6 versus 5.6). Interestingly, no significant decrease was observed with laser treatment at day 7 compared to controls (mean, 18.1 versus 20.5). A significant decrease in the NP area was observed only at day 90 compared to the control condition (mean, 5.6 versus 20.4).

At the same time, Boos' scoring allowed us to compile and quantitatively assess degenerative tissue changes (Figure 5(b)). In the control condition, no significant increase was observed. Conversely, an increase in both enzyme and laser conditions was observed as a function of time. Interestingly, Boos' scoring after laser treatment increased more progressively compared with the enzyme treatment, with values of approximately 9.5 and 13.5 at day 7 and 17.5 at day 90, respectively.

## 4. Discussion

To clinically address LBP originating from IVD degeneration (discogenic lumbalgia), tissue engineers have sought to develop cell-based approaches to functionally “regenerate” the damaged tissue [4]. To preclinically test the relevance of these cell-based approaches, animal models are required. Numerous models of degenerative disc disease have been developed. Whereas some of them are instrumental for studying the specific aspects of IVD biology or pathology, most of them fail to meet all the criteria required for the preclinical testing of IVD regenerative medicine approaches. These models are either spontaneous or experimentally induced models.

Spontaneous models use sand rat, Chinese hamster, transgenic mice, or rats [28–31] as well as nonchondrodystrophic or chondrodystrophic canine breeds [32–34]. While these models are of considerable value to study the IVD's physiopathological mechanisms, they generally remain too small to develop strategies that could be easily transferred to the human clinical situation. Back pain is well documented in the dog with a high prevalence of spontaneous disc hernia. However, the veterinary patients are difficult to recruit for large-scale studies in part because of the heterogeneity of the breeds and difficulties to obtain owner consent. In addition, such canine model remains problematic to implement because it implies exogenous (mechanical stress and nutritional effects) and endogenous (genetic predisposition) factors that can be difficult to control. Moreover, nonchondrodystrophic breeds develop DDD quite late in their lifespan making them inappropriate as an experimental model.

Among experimentally induced animal models, three modalities to induce IVD degeneration have been defined. First, mechanical models based on the application of overloading stimuli on the IVD using an axial dynamic loading system [7], lumbar fusion [8], tail suspension [9], torsion [10], compression [6, 11], bipedism [12], muscle stimulation [13], and resection of spinal process or facet joint [14] have been described. As spontaneous models, they are essentially suitable for physiopathological studies. Their reproducibility is generally reported to be low and they often require invasive surgical protocols that remain debatable from an ethical point of view.

Second, physical models (needle puncture [16, 35], scalpel stab of AF [15], end plate injury [36], and nucleus aspiration [16, 37–43]) have been developed in numerous species including rabbit [35], sheep [44], or pig [45, 46]. Unfortunately, aspiration and scalpel lesion have proved to be poorly reliable because of difficulties in inducing accurate and reproducible lesions to the disc. Needle puncture lesion was generally found more gradual and reproducible but tissue lesions were reported to substantially dependent on the gauge size of the needle used.

Moreover, the IVD damage induced by aspiration is quite far from spontaneous degeneration and could instead be considered as a model of disc herniation because of the removal of the cells and extracellular matrix from the center of the disc.

Third, chemical models were widely studied in numerous species such as rats [24], dogs [20, 22, 23], and even cattle [19, 21]. Hyaluronidase [17], chondroitinase ABC [23], and papain [18] were the most widely used enzymes. All of them deteriorated the extracellular matrix components, notably proteoglycans, and they were considered in evaluation of regenerative strategies of the IVD. The reliability of IVD degeneration is widely debated and the main limitation is the persistence of proteolytic enzymes, metabolites, and degradation factors that could be cytotoxic not only for IVD resident cells, but also for the injected regenerative cells. Finally, since the optimal dose of enzyme has not been clearly established, enzyme injection has usually been associated with rapid degenerative changes that fail to completely mimic the naturally occurring DDD.

Considering these animal models, new research has focused on the potential of an innovative laser treatment to induce a reliable, progressive, large, and manageable IVD degeneration model [25]. In a preliminary study, the influence of the laser treatment on IVD degeneration was compared with NP aspiration (a mechanically induced model). The results revealed the consistency of this innovative experimentally induced animal model of IVD degeneration. To provide a model suitable for preclinical assessment of the early stages of IVD degeneration, we now sought to precisely analyze the progressivity of the degeneration induced by laser. The results demonstrated that the enzyme induced a quick degeneration of the IVD with a drastic decrease of the T2wsi and IVD height. Tissue integrity was also altered regarding the decrease of the NP area measurement and a high Boos' scoring value at early times, despite the absence of a clearly delimited boundary between NP and AF. Interestingly, the laser procedure induced a progressive degenerative process characterized by a slow decrease in the T2wsi and IVD height as shown by MRI and X-ray, respectively. This slow and moderate decrease in IVD height is much closer to that observed in spontaneous degeneration in humans. Indeed, in humans, aging induces an approximately 11% decrease in the lumbar IVD height after 15 years in follow-up studies [47]. The histological data and the elevated Boos' scoring also confirmed the progressive alteration of the NP extracellular matrix. The exact mechanisms by which laser irradiation induces IVD degeneration remain poorly understood but are likely to involve the high temperature resulting from the irradiation, as reported previously to explain the changes in ultrastructure and composition observed in laser-irradiated dentin [48].

Like other models, this one has a number of limitations that deserve consideration. The rabbit NP is populated with notochordal cells rather than the chondrocytic cells found in human IVD and the role of these cells has not yet been clarified. Another animal model should be tested to confirm the results obtained in rabbits in the present study. Nevertheless, this model has several advantages over other existing models. First, it is extremely simple and inexpensive, requiring only percutaneous access and a minimal surgical procedure as well as shorter anesthesia, thus allowing faster recovery. Second, the model is extremely effective, given that several degenerative IVDs can be produced per animal. One of the most promising features of this model is that it yields significant changes in the IVD treated that correlate with the histological changes classically described during DDD in humans. This process seems more progressive and therefore closer to spontaneous aging than the process observed in the nonprogressive tissue damage triggered by enzyme treatment. Therefore, the laser model can be considered as an interesting model to investigate the safety and efficacy of preventive treatment of disc disease because it allows us to intervene early in the course of the disease. Moreover, it allows inducing reproducible tissue lesions without the risk of external remnants and the routes tested on these models could be easily transposed to future human applications.

## 5. Conclusion

This study shows that progressive IVD degeneration can be induced in the rabbit by a laser procedure. MRI, radiological, and histological data have confirmed the relevance of this experimentally induced animal model of IVD degeneration compared to an enzymatic procedure (chemonucleolysis). This model could be quite instrumental in helping validate a cell-based strategy for the prevention of IVD degeneration.

## Figures and Tables

**Table 1:** Group organization and treatments protocol.

		Group 1				Group 2					Group 3		
	Rabbit number 1	Rabbit number 2	Rabbit number 3	Rabbit number 4	Rabbit number 5	Rabbit number 6	Rabbit number 7	Rabbit number 8	Rabbit number 9	Rabbit number 10	Rabbit number 11	Rabbit number 12	Rabbit number 13
IVD L2-L3	CTRL	CTRL	CTRL	CTRL	Laser	Laser	E	E	CTRL	Laser	Laser	E	E

IVD L3-L4	CTRL	CTRL	CTRL	Laser	Laser	E	CTRL	Laser	Laser	Laser	E	CTRL	Laser

IVD L4-L5	CTRL	CTRL	CTRL	E	E	CTRL	Laser	Laser	E	E	CTRL	Laser	Laser

IVD L5-L6	CTRL	CTRL	CTRL	E	CTRL	E	Laser	E	E	CTRL	E	Laser	E

MRI and X-ray	D0	Rabbit 2: days 0 and 7				D0 and D7					D0, D7, D30, D60, and D90		
		Rabbit 3: days 0, 7, 30, 60, and 90											

Histology	Sacrifice at day 0	Rabbit 2: sacrifice at day 7				Sacrifice at day 7					Sacrifice at day 90		
		Rabbit 3: sacrifice at day 90											

CTRL: control; E: enzyme; IVD: intervertebral disc.
